# Novel Interleukin-10 Gene Polymorphism Is Linked to Gestational Diabetes in Taiwanese Population

**DOI:** 10.3389/fgene.2019.00089

**Published:** 2019-02-18

**Authors:** Jessica Kang, Chia-Hsiung Liu, Chien-Nan Lee, Hung-Yuan Li, Chien-Wen Yang, Shu-Chien Huang, Shin-Yu Lin, Tzuu-Shuh Jou

**Affiliations:** ^1^Department of Obstetrics and Gynecology, National Taiwan University Hospital, Taipei, Taiwan; ^2^Department of Surgery, College of Medicine, National Taiwan University, Taipei, Taiwan; ^3^Department of Internal Medicine, National Taiwan University Hospital, Taipei, Taiwan; ^4^Graduate Institute of Molecular Medicine, College of Medicine, National Taiwan University, Taipei, Taiwan; ^5^Department of Surgery, National Taiwan University Hospital, Taipei, Taiwan; ^6^Graduate Institute of Clinical Medicine, College of Medicine, National Taiwan University, Taipei, Taiwan; ^7^Center of Precision Medicine, College of Medicine, National Taiwan University, Taipei, Taiwan

**Keywords:** gene polymorphism, gestational diabetes, interleukin-10, single nucleotide polymorphism, inflammation

## Abstract

**Objective:** The association of interleukin-10 (IL-10) polymorphism with diabetes and its complication was recently established, while there were few researches considering the potential role of IL-10 in gestational diabetes (GDM). This study aimed to investigate the association between IL-10 gene rs1800896 (−1082 A/G), rs1800871 (−819 T/C), rs1800872 (−592 A/C), and rs3021094 (3388 A/C) single nucleotide polymorphisms (SNPs) and GDM susceptibility.

**Methods:** This study included 72 GDM patients and 100 healthy pregnant women. Direct sequencing of the products from polymerase chain reactions of the extracted genomic DNA from study subjects were conducted for analyzing IL-10 gene polymorphism and further genotype frequencies were compared. Plasma IL-10 concentration was measured by ELISA method.

**Results:** The results revealed no significant difference in −592 A/C, −819 T/C, and −1082 A/G genotypes. Significantly increased prevalence of A allele (*P* = 0.028, OR = 1.69, 95% CI = 1.081–2.64) and A/A genotype (*P* = 0.031, OR = 2.881, 95% CI = 1.145–7.250) at a previously un-characterized rs3021094 SNP were discovered in the GDM group. Increased IL-10 levels and insulin resistance were also related to the genotype of rs3021094. The risk of GDM was increased when IL-10 level was over 6.5 pg/ml.

**Conclusion:** Our study demonstrated that A allele and A/A genotype of rs3021094 SNP in IL-10 gene were linked to increased risk for GDM, IL-10 plasma level and insulin resistance, which could be potential targets for early screening and detection of GDM.

## Introduction

Gestational diabetes (GDM) is defined as impaired glucose tolerance developed during pregnancy, and it has become the most common metabolic disorder in pregnant women (American Diabetes Association, 2018). It would not only increase the risk of adverse maternal and fetal outcomes, but also the risk of developing future diabetes in both mother and their offspring (Chiefari et al., 2017). The relationship between chronic status of inflammation, insulin resistance, and type 2 diabetes is well-established (Wellen and Hotamisligil, 2005; Olefsky and Glass, 2010). Given the sharing of pathogenic contribution from common genetic factors between type 2 DM and GDM (Robitaille and Grant, 2008), we deduce that dysregulation of immune regulators which contribute to the onset of type 2 DM may also play an important role in GDM. While multiple cytokines are involved in the dysregulation of the immune response which is implicated in the pathogenesis of type 2 DM (Hu et al., 2004; Al-Shukaili et al., 2013), CRP (Wolf et al., 2003) and IL-10 (Atègbo et al., 2006) are the few cytokines discovered to be associated with GDM.

Interleukins are a variety of signaling molecules that regulate the function of human immune system (Lippitz, 2013). The genetic polymorphisms of interleukins are closely related to their activity, presumably through altering cytokine function, or dysregulating their expression. IL-10 is an important anti-inflammatory cytokine that plays a crucial role as an immune response modulator. The encoding gene of IL-10 is located on chromosome 1 (1q31–1q32) (Eskdale et al., 1997), and IL-10 is produced by activated T cells, monocytes, and B cells (Chagas et al., 2013; Jin et al., 2013). IL-10 production is related to genetic variations in its promoter region (Westendorp et al., 1997), and this region controls transcription and contains SNPs that are related to diabetes and its complication (Turner et al., 1997; Tegoshi et al., 2002; Mohebbatikaljahi et al., 2009; Mahmoud et al., 2016). The relationship between IL-10 concentration and GDM has yet to be concluded, while both decreased (van Exel et al., 2002) and increased IL-10 levels have been reported in type 2 DM and GDM patients (Atègbo et al., 2006; Al-Shukaili et al., 2013).

The polymorphic sites within the promoter region of IL-10 include several SNPs [rs1800896 (−1082 A/G), rs1800871 (−819 T/C), and rs1800872 (−592 A/C)], which have been described in relation to diabetes, and two microsatellite loci in the 4 kb immediately upstream of the human IL-10 transcription initiation site (Turner et al., 1997; Eskdale et al., 1998). *In vitro* work using peripheral blood mononuclear cells has suggested that −1082G, −819C, and −592C alleles are associated with higher IL-10 production (Turner et al., 1997; Edwards-Smith et al., 1999). In contrast to a lack of association of these three promoter SNPs in type 1 DM population (Tegoshi et al., 2002; Urcelay et al., 2004; Mohebbatikaljahi et al., 2009), the relationship between SNPs −1082 A/G and −592 A/C and type 2 DM has been detected in several ethnic groups (Helaly et al., 2013; Saxena et al., 2013; Bai et al., 2014). Besides, an association with DM nephropathy has also been detected in these three polymorphic sites (Ezzidi et al., 2009; Mahmoud et al., 2016). As for GDM, one recent study has demonstrated that SNP −592 is associated with the development of GDM but irrelevant with serum IL-10 level (Montazeri et al., 2010), while another case-control study reported no significant associations between SNP −1082 and GDM (Gueuvoghlanian-Silva et al., 2012). Although another less characterized IL-10 gene SNP rs3021094 (3388 A/C) at the intron region has been reported to show a weak association with diabetic nephropathy in Chinese population (Zhou et al., 2016), the significance of this SNP has not been established with other diseases previously.

Since inflammation is implicated in the pathogenesis of GDM, we hypothesize that IL-10 polymorphisms and its serum level may be linked to hyperglycemia during pregnancy. In this study we established the association of a novel IL-10 gene polymorphism (rs3021094) presumably through modulating the levels of IL-10 expression in GDM of Taiwanese population.

## Materials and Methods

### Patients

This is a retrospective study carried out in the Department of Obstetrics and Gynecology of National Taiwan University Hospital, Taipei, Taiwan. Total 172 Taiwanese singleton pregnant women (100 healthy control and 72 cases with GDM) were recruited from January 2015 to Feb 2018. All the study subjects are consecutive and unrelated. The exclusion criteria were as the following: multiple gestation, preeclampsia, pregnancy induced hypertension, autoimmune disease, previous polycystic ovary syndrome, pre-existing diabetes, and hypertension. Control subjects were healthy pregnant women without any maternal or fetal disorder, who were recruited at the same obstetrics clinic as the GDM group. The diagnosis of GDM was based on 2010 International Association of the Diabetes and Pregnancy Study Groups (IADPSG) guideline during 24 weeks to 28 weeks of gestation (IADPSG, 2010). The control group has no case with previous GDM, while the GDM group has 19 cases with previous GDM. We referred all of these GDM women to dietitian and metabolic physician. They received diet control for 2 weeks, and fasting glucose was rechecked again at appointed follow-up clinic. If blood glucose levels were normalized, then the GDM subjects would be advised to continue diet control. If not, hypoglycemic medication such as metformin, would be prescribed. Four GDM patients of this study received hypoglycemic medication (three received oral metformin and one received additional subcutaneous insulin injection). Fasting whole blood samples were collected for four times (first trimester, second trimester, and third trimester) in order to analyze basic biochemical data including fasting glucose, HbA1c, and lipid profiles during the pregnancy course. Another 3 ml of maternal venous blood was drawn from each enrolled case upon admission before delivery for polymorphisms study. Clinical information of the newborns including gestational age at birth, birth body weight, and head circumference were recorded by the attending pediatricians and nurses after birth.

Sample size estimation was based on our preliminary data of IL-10 SNP rs3021094 (3388 A/C) gene polymorphism frequency. Applying allele risk data in STATA 14 (Stata Crop, College Station, United States) and considering an 80% power and a two-tailed alpha of 0.05, a sample size of 71 patients and 81 participants in control group would be enough to detect an association between the alleles and GDM.

All cases were followed from the first trimester of pregnancy to 6 weeks postpartum. Informed consent was obtained from each study subject after the nature of the study was fully explained, and the study was approved by the Ethics Review Committee of National Taiwan University Hospital, Taipei, Taiwan (201412216RINA).

### Extraction of Genomic DNA

Maternal venous blood was collected in EDTA-treated tubes at delivery and prepared for buffy coat specimens. Genomic DNA was extracted from maternal buffy coat using DNeasy Blood & Tissue kit according to the manufacturer’s instructions (QIAGEN GmbH, Germany)/QIAamp DNA Micro kit (Qiagen Inc.).

### Genotyping for Genetic Variants

A set of primer pairs (Table 1) was designed with Primer3 (Koressaar and Remm, 2007) to amplify the 2 kb promoter region of IL-10 gene. The polymerase chain reaction (PCR) products were purified by ExoSAP-IT (GE Healthcare, United States) followed by sequencing reactions using the BigDye Terminator v3.1 Cycle Sequencing Kit (Thermo Fisher Scientific Inc./Applied Biosystems, United States). The reaction products were purified and run on a 3730xl DNA Analyzer (Applied Biosystems). The variations of the IL-10 gene were detected by Geneious version 8.0.5 (www.geneious.com) (Kearse et al., 2012).

**Table 1:** The sequence of primers used to generate polymerase chain reaction products for identifying the interleukin 10 genotypes among the individual subjects.

SNP	Primer sequences	PCR product size (bp)
rs1800872 (−592)	Forward: 5′-GGTGAGCACTACCTGACTAGC-3′	681
	Reverse: 5′-AAAGCCACAATCAAGGTTTCCC-3′	
rs1800871 (−819) and rs1800896 (−1082)	Forward: 5′-CAGGGAGGATGAGTGATTTGC-3′	786
	Reverse: 5′-GGATTCTCAGGCACATGTTTCC-3′	
rs3021094 (3388)	Forward: 5′-GAATCCTAGATCAAGCCATGGG-3′	680
	Reverse: 5′-TGGGGAAATAACTGAAATGCGG-3′	

### Enzyme-Linked Immunosorbent Assays

Whole blood samples were collected in EDTA tubes, centrifuged at 930 × *g* for 5 min, and the supernatants were aliquoted and stored at −80°C. The IL-10 concentrations of these plasma samples were measured with BD OptEIA Human IL-10 ELISA Kit II (BD Biosciences Pharmingen, California, United States) according to the manufacturer’s instructions.

### Statistical Analysis

The data were analyzed using SPSS 22.0 (SPSS, Chicago, United States). Data analysis started with descriptive statistics, including means and standard deviation for continuous variables. Mann-Whitney U test was used to compare the significant differences in patient characteristics between the GDM and the control groups. *P*-values of <0.05 were considered significant.

The frequencies of IL-10 gene alleles and genotypes in different groups were compared using the chi-squared test or Fisher’s exact test. The odds ratios (OR) and 95% confidence intervals (CI) were calculated using logistic regression with age as a covariate. Kruskal–Wallis *H* test and Mann Whitney U test were used to evaluate the associations of the insulin resistance and IL-10 plasma levels in subjects with different IL-10 genotypes. Plasma IL-10 concentrations in different groups were presented as medians with quartile differences in figures. Receiver-operating characteristic (ROC) analysis was performed to establish the predictive ability of IL-10 concentration for GDM and find out the cut-off value by Youden index.

## Results

### Patient Characteristics

Maternal and newborn characteristics of all 172 cases are shown in Table 2. Four GDM patients received hypoglycemic medication (three received oral metformin and one received subcutaneous insulin injection), while other GDM patients received diet control without hypoglycemic medications. The GDM women were older than the control cohort. The first trimester fasting glucose, first and third trimester HbA1c, and the plasma glucose levels post-75 g oral glucose tolerance test (OGTT) were significantly higher in the GDM group during the period of pregnancy. However, no significant difference was observed in the lipid profiles except for first and third trimester triglyceride level between GDM and control subjects. Overall, the GDM women delivered at earlier gestational age, and the head circumference among the newborns was relatively smaller than the control group, which might be due to diet control and early labor induction of the GDM group. There was no significant difference in birth body weight and body height of the newborns.

**Table 2:** Comparing of baseline characteristics of GDM patients with the control group.

	GDM (*n* = 72)	Normal (*n* = 100)	
	Mean *(SD)*	Mean *(SD)*	*P*-value
**First trimester**			
Age (years)	34.7 (4.2)	32.8 (3.6)	0.006
Body weight (kg)	58.4 (10.0)	56.7 (9.1)	0.212
BMI (kg/m^2^)	22.6 (3.4)	22.1 (3.6)	0.124
Fasting glucose (mg/dL)	86.5 (6.9)	80.6 (4.3)	<0.001
HbA1c (mmol/mol)	36 (4.5)	33 (2.5)	0.004
HbA1c (%)	5.4 (0.4)	5.2 (0.2)	0.004
Fasting cholesterol (mg/dL)	185 (38.4)	175.5 (32.2)	0.284
Fasting triglyceride (mg/dL)	120.5 (47.6)	102.1 (41.4)	0.009
Fasting LDL (mg/dL)	98.2 (30.0)	92.5 (26.2)	0.287
Fasting HDL (mg/dL)	70.2 (18.0)	71.2 (12.8)	0.484
**Second trimester**			
Fasting glucose (mg/dL)	84.5 (11.1)	77.3 (4.5)	<0.001
1 h glucose post-OGTT (mg/dL)	169.7 (34.6)	122.5 (25.1)	<0.001
2 h glucose post-OGTT (mg/dL)	152.1 (28.2)	108.2 (18.9)	<0.001
HbA1c (mmol/mol)	31 (4.5)	30 (5.5)	0.171
HbA1c (%)	5 (0.4)	4.9 (0.5)	0.171
Fasting cholesterol (mg/dL)	241.7 (46.8)	242.2 (37.9)	0.851
Fasting triglyceride (mg/dL)	196.6 (73.4)	177.1 (63.6)	0.114
Fasting LDL (mg/dL)	139.2 (40.9)	140.7 (34.2)	0.659
Fasting HDL (mg/dL)	82.5 (19.9)	85.1 (15.6)	0.149
Third trimester			
Body weight (kg)	67.4 (10.8)	66.3 (8.1)	0.885
Fasting glucose (mg/dL)	86.1 (19.3)	82.3 (14.7)	0.369
HbA1c (mmol/mol)	37 (4.5)	36 (2.5)	0.002
HbA1c (%)	5.5 (0.4)	5.4 (0.2)	0.002
Fasting cholesterol (mg/dL)	270.5 (51.2)	271.4 (51.4)	0.830
Fasting triglyceride (mg/dL)	334.7 (105.7)	292.3 (115)	0.038
Fasting LDL (mg/dL)	132.5 (37.0)	140 (40.6)	0.366
Fasting HDL (mg/dL)	82.8 (21.2)	80 (14.8)	0.730
**Newborn**			
Gestational age at birth (weeks)	38.4 (2.0)	39.2 (1.3)	0.003
Body height (cm)	48.9 (2.3)	49.3 (2.0)	0.256
Birth weight (grams)	2987 (525.8)	3104 (386.9)	0.082
Head circumference (cm)	33.4 (1.6)	33.9 (1.4)	0.019
Chest circumference (cm)	31.6 (1.7)	32.1 (1.6)	0.058

### IL-10 Gene Variants

Three common SNPs in the IL-10 promotor region (−1082 A/G, −819 T/C, and −592 A/C) were evaluated in this study by direct sequencing of the PCR product amplified by primers designed to cover these SNP sites, and the result showed no significant difference in either the genotype or allele frequencies between the GDM and control groups (Table 3). However, examination of a previously less characterized SNP (rs3021094) in an intron of the IL-10 gene showed an interesting finding.

**Table 3:** The relationship between different genotypes and alleles of IL-10 gene SNPs (rs1800872, rs1800871, rs1800896, and rs3021094) and their susceptibility to GDM was analyzed by co-dominant model.

SNP	Genotype/Allele	Control (*N* = 100) *n* (%)	Case (*N* = 72) *n* (%)	*P* value	OR (95% CI)
rs1800872 (−592)	AA	51 (51.0)	33 (45.8)	Ref.	1
	AC	39 (39.0)	32 (44.5)	0.515	1.252 (0.650–2.411)
	CC	10 (10.0)	7 (9.7)	1.000	1.146 (0.384–3.421)
	A	141 (70.5)	98 (68.1)	0.637	1.139 (0.707–1.836)
	C	59 (29.5)	46 (31.9)		
rs1800871 (−819)	TT	49 (49.0)	33 (45.8)	Ref.	1
	TC	41 (41.0)	32 (44.5)	0.745	1.148 (0.597–2.207)
	CC	10 (10.0)	7 (9.7)	1.000	1.102 (0.369–3.289)
	T	139 (69.5)	98 (68.1)	0.814	1.087 (0.676–1.748)
	C	61 (30.5)	46 (31.9)		
rs1800896 (−1082)	AA	88 (88.0)	64 (88.9)	Ref.	1
	AG	12 (12.0)	8 (11.1)	1.000	0.872 (0.327–2.323)
	GG	0 (0.0)	0 (0.0)	N.A.^†^	N.A.
	A	188 (94.0)	136 (94.4)	1.000	1.137 (0.440–2.938)
	G	12 (6.0)	8 (5.6)		
rs3021094 (3388)	AA	24 (24.0)	26 (36.1)	0.031	2.881 (1.145–7.250)
	AC	48 (48.0)	35 (48.6)	0.164	1.776 (0.770–4.094)
	CC	28 (28.0)	11 (15.3)	Ref.	1
	A	96 (48.0)	87 (60.4)	0.028	1.690 (1.081–2.640)
	C	104 (52.0)	57 (39.6)		

*^†^Non-available.*

The rs3021094 A allele frequency significantly increased in the GDM group (*P* = 0.028, OR = 1.69, 95% CI = 1.081–2.64) (Table 3). Significant association of GDM was noted with A/A genotype verse C/C genotype (*P* = 0.031, OR = 2.881, 95% CI = 1.145–7.250), while no association was noted with A/C verse C/C genotype (*P* = 0.164). Taken together, these results indicated that A allele and A/A genotype in this genetic locus were associated with GDM.

### Insulin Resistance Evaluation

**FIGURE 1:**
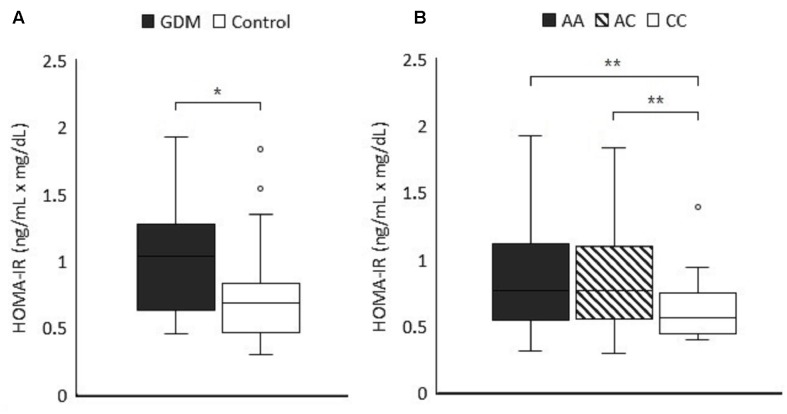
**(A)** The first trimester HOMA-IR values (ng/mL × mg/dL) were measured in 24 GDM patients and 65 control subjects (*P* = 0.004). **(B)** First trimester HOMA-IR values in cases of Figure 1A were categorized according to the different genotypes of rs3021094 SNP (*n* = 25 for A/A, 42 for A/C, and 22 for C/C). Data represented the median and interquartile range of each group. Statistical differences between groups were analyzed by Kruskal–Wallis *H* test and Dunn’s test. *^∗^P* < 0.05, ^∗∗^*P* < 0.01.

C-peptide of 89 subjects (65 controls and 24 GDM patients) were available for HOMA-IR calculation to evaluate insulin resistance (Wallace et al., 2004) which was significantly higher in the GDM group (median level 1.03 vs. 0.68 ng/mL × mg/dL, *P* = 0.004 by non-parametric test) (Figure 1A). The comparison of HOMA-IR in different genotypes of rs3021094 (A/A, A/C, and C/C) using Mann Whitney U test also revealed significant difference between groups (Overall *P*-value for Kruskal-Wallis test = 0.018, *P*-value for Mann Whitney U test of A/A vs. C/C = 0.003, A/C vs. C/C = 0.005) (Figure 1B). These data demonstrated increased insulin resistance in population with allele A at rs3021094 SNP, and this result was compatible with our earlier finding that subjects with allele A at this locus carried a higher risk of GDM.

### IL-10 Plasma Levels

**FIGURE 2:**
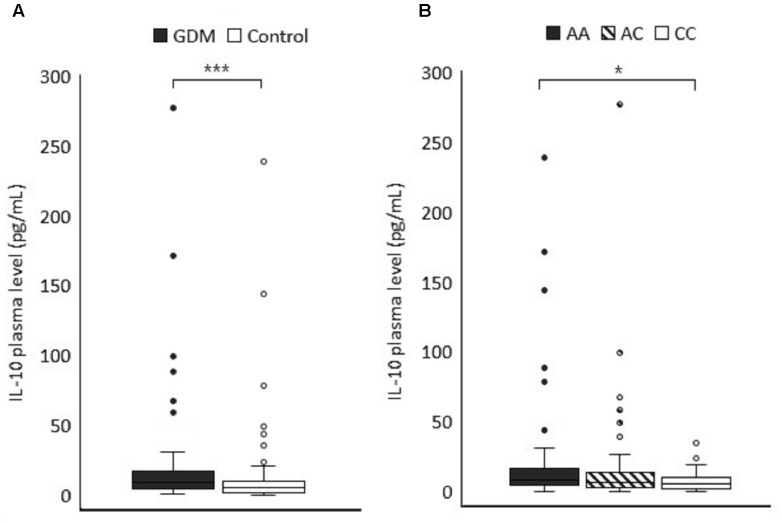
**(A)** The plasma IL-10 level (pg/mL) was compared between GDM and control subjects (*P* < 0.001). **(B)** The IL-10 levels of the study subjects were categorized according to the genotypes at the rs3021094 SNP locus (3388A/C). Data represented the median and interquartile range of each group. Statistical differences between groups were analyzed by Kruskal–Wallis *H* test and Dunn’s test.*^∗^P* < 0.05, ^∗∗∗^*P* < 0.001.

**FIGURE 3:**
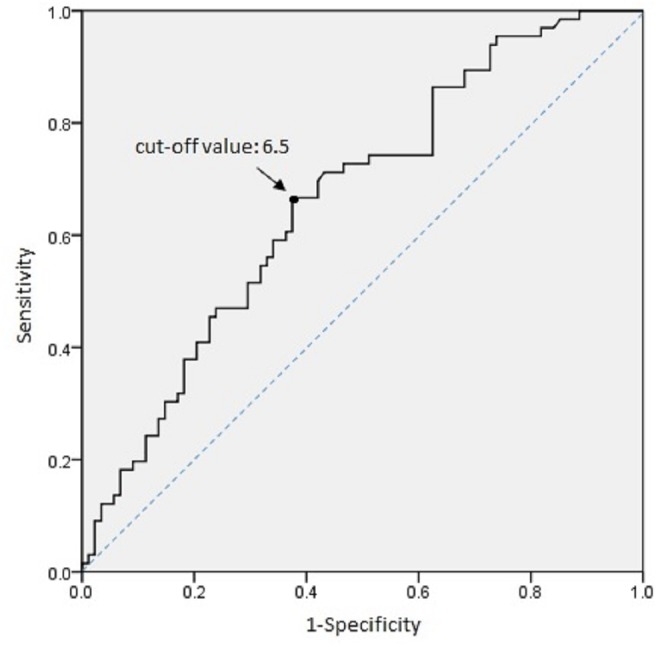
Optimal cut-off IL-10 concentration for predicting GDM risk was established by ROC analysis. Increased risk of GDM was noted in population with IL-10 concentrations above 6.5 pg/ml.

To further explore the functional significance of IL-10 polymorphism in GDM pathogenesis, the plasma IL-10 concentration of 66 GDM patients and 89 normal pregnant women whose IL-10 gene polymorphism had been genotyped in this study was quantified using ELISA. The plasma IL-10 levels were significantly higher in GDM women than the controls (median 8.31 vs. 5.32 pg/mL, *P* < 0.001 by non-parametric test) (Figure 2A). ROC analysis was used to identify the optimal cut-off level of plasma IL-10 for predicting GDM (the area under the ROC curve = 0.665, 95% CI = 0.579–0.75, *P* < 0.001) (Figure 3). Subjects with IL-10 levels higher than 6.5 pg/mL had greater risk for GDM than those with the IL-10 levels below this cut-off value (*P* < 0.001, OR = 3.571, 95% CI = 1.82–7.009).

We also compared the associations between plasma IL-10 concentration and different genotypes of −1082A/G, −819 T/C, −592 A/C, and rs3021094 SNPs, respectively. The associations were not significantly related to the three common SNPs. However, subjects with A/A genotype at the locus of rs3021094 had higher IL-10 levels compared with those carrying C/C (Overall *P*-value for Kruskal-Wallis test = 0.101; *P*-value for Mann Whitney U test of A/A vs. C/C = 0.024) (Figure 2B).

## Discussion

The underlying mechanism of GDM remains undetermined. Although accumulating evidences have implied a relationship between DM and gene polymorphism, there have been limited studies investigating genetic predisposition of GDM. We therefore explore the genetic polymorphisms of Taiwanese GDM cases in order to distinguish candidate characteristics in our population.

Reviewing previous medical history of our study subjects, there was no case diagnosed with GDM in the control group, while 19 women were diagnosed as GDM during their previous pregnancy in the GDM group of current study. Thirty-three of the total 72 GDM patients received 75 g OGTT again postpartum during follow-up. Three women were diagnosed as impaired glucose tolerance and none as DM. Five women with poorly controlled blood sugar were followed-up at the outpatient department of Endocrinology and Metabolism in National Taiwan University Hospital. Five of nine women were diagnosed as GDM during their subsequent pregnancy, including four under diet control and one using anti-diabetic medication. Longer following-up time is necessary to evaluate the long-term development of diabetes in our study cases. According to the literature, there is an increased subsequent GDM risk after being diagnosed as GDM at their first pregnancy. It would be intriguing to observe whether the IL-10 gene polymorphism might affect the progression of initial GDM to onset of subsequent GDM or full blown DM.

Genetic polymorphism in IL-10 is of great clinical interest because IL-10 is an important cytokine which regulates inflammatory and immune responses in various pathological situations including diabetes (Helminen et al., 1999; Jin et al., 2013; Bai et al., 2014). Our study is the first of related studies designed to focus on GDM population. Most of the previous IL-10 polymorphism studies are aiming on type 1 DM, type 2 DM, or diabetic related complications. For example, no significant difference of IL-10 genotype and allele frequencies has been discovered between controls and type 1 DM in Turkish, Japanese, or French population (Tegoshi et al., 2002; Urcelay et al., 2004; Mohebbatikaljahi et al., 2009). Another study demonstrates that IL-10 genetic polymorphisms has only limited contribution to disease susceptibility in French type 1 DM patients (Reynier et al., 2006). Therefore, the SNP polymorphism in IL-10 promoter region might not be an ideal marker in the routine genetic screening of high-risk individuals for type 1 DM.

Different ethnic backgrounds can apparently modulate the impacts of IL-10 gene polymorphism on glucose homeostasis contributed by these different genotypes. For instance, the −1082 G/G genotype in Egyptian subjects have an increased risk for type 2 DM (Helaly et al., 2013), while no association has been discovered between positions −1082 and −592 genetic variants with diabetes in Caucasian Italian Subjects (Scarpelli et al., 2006). The subjects carrying the −1082 G/G and −592 A/A genotype have been demonstrated to carry a significantly increased risk of type 2 DM in Chinese population (Bai et al., 2014). Furthermore, another independent study demonstrates that more type 2 DM subjects carry −592C and −819C alleles in Taiwanese population, and those type 2 DM patients who carry these alleles are associated with high levels of IL-10 production (Chang et al., 2005).

As for the relationship between diabetic complications and IL-10 polymorphism, IL-10 −592 genotype frequency, and allele frequency are significantly different in the diabetic patients with nephropathy (Kung et al., 2010; Mahmoud et al., 2016). IL-10 −1082 G/G polymorphism has also been found to be associated with the susceptibility to diabetic neuropathy in Indian population (Ramesh et al., 2014). Neither the −1082 A/G nor the −592 A/C polymorphism is associated with diabetic nephropathy in Tunisian (Ezzidi et al., 2009). Interleukin-10 (IL-10) level *per se* is found significantly elevated in diabetic nephropathy patients with association to IL-10 −592 C/C genotype (Mahmoud et al., 2016).

Previous studies have proved that the distribution of alleles and genotypes frequencies of IL-10 −819 T/C and −592 A/C are strongly linked (Zeng et al., 2009; Naib et al., 2013). The result of our study also showed such a relationship (Table 3). However, there was also no significant susceptibility of GDM over both SNPs.

There is very few information about the role of IL-10 gene polymorphism in GDM. The only related study has been conducted in Malaysia, which shows allelic genotypes that SNP at position −597 (which is equivalent to −592 in our study) are significantly different between GDM and control group. In contrast to our finding, they did not detect any difference in IL-10 plasma levels during pregnancy among subjects carrying different IL-10 gene variants (Montazeri et al., 2010). Although our study also revealed no significant contribution in IL-10 levels from all three common SNPs (−592, −819, and −1082) at IL-10 gene promoter, gene variants at an IL-10 intron SNP rs3021094 showed a distinct influence in affecting IL-10 plasma levels.

The rs3021094 (3388 A/C) is an intron SNP located on 1q32.1. Previous population genetics study shows the allelic frequency is about 85% for A, and is about 15% for C (Sherry et al., 2001). Although one prior study has investigated a weak association between IL-10 rs3021094 SNP site and DM nephropathy (Zhou et al., 2016), our study was the first to report a connection between IL-10 rs3021094 allele A and GDM susceptibility. Furthermore, our study also revealed an increased insulin resistance and IL-10 levels in A/A + A/C genotypes.

Previous study has reported that polymorphisms of the IL-10 gene promoter is correlated to *in vitro* IL-10 production, which have been demonstrated to be genetically predisposed (Turner et al., 1997; Eskdale et al., 1998). Another research studying the IL-10 genotype and hepatitis C also shows strong association of different promoter haplotypes with degrees of IL-10 production (Edwards-Smith et al., 1999). Thus we hypothesized that genetic variation may influence IL-10 production and set up to evaluate the association between different SNP genotypes and IL-10 levels in the GDM patients. Although no correlation was noted between different genotypes of the three common IL-10 promoter SNPs and IL-10 concentration, increased IL-10 production together with more GDM incidence were found in A/A genotype of rs3021094 comparing to C/C genotype. We also discovered increased insulin resistance in population with allele A. These findings implied that allele A of rs3021094 might increase IL-10 production and GDM susceptibility.

Our study also demonstrated that IL-10 concentration was significantly higher in GDM than control group. A significantly increased risk of GDM was noted when IL-10 concentration was over 6.5 pg/ml. The result suggested that GDM development is related to cytokine IL-10 production which could lead to an immune status that causes metabolic derangement. This finding was surprising and seemingly contradictive to a previous report that higher plasma IL-10 levels are associated with a better pregnancy rate among women who undergo *in vitro* fertilization programs (Wu et al., 2001). IL-10, as a potent anti-inflammatory cytokine, has been postulated to facilitate maternal immune tolerance to the accommodation of the fetoplacental unit in human pregnancy (Hanna et al., 2000). One of the possible explanation for the noted higher IL-10 levels in GDM than the normally pregnant women is because more IL-10 is called upon to compensate an already “stressed” fetoplacenta unit. It would be therefore interesting to correlate the IL-10 levels in our study subjects to those proinflammatory cytokine concentrations.

In summary, we reported a novel connection between IL-10 rs3021094 SNP with GDM incidence, IL-10 level, and insulin resistance were also in significant correlation. Such findings could imply a pathogenic role of IL-10 in GDM and provide further guidance for future investigation.

## Author Contributions

S-YL, C-NL, H-YL, S-CH, and T-SJ conceived the idea and presided over the conduction of the study. JK, S-YL, and T-SJ wrote the main manuscript text. C-HL, JK, C-WY, and S-CH performed statistical analyses and prepared Figure 1–3 and Tables 1–3.

## Conflict of Interest Statement

The authors declare that the research was conducted in the absence of any commercial or financial relationships that could be construed as a potential conflict of interest.
